# Beyond the Biomarker: Unveiling the Multifaceted Role of Osteopontin in Both Physiological and Pathological Processes

**DOI:** 10.3390/biomedicines12050982

**Published:** 2024-04-30

**Authors:** Davide Raineri, Annalisa Chiocchetti, Giuseppe Cappellano

**Affiliations:** 1 Department of Health Sciences, Interdisciplinary Research Center of Autoimmune Diseases-IRCAD, Università del Piemonte Orientale, 28100 Novara, Italy; davide.raineri@uniupo.it (D.R.); annalisa.chiocchetti@med.uniupo.it (A.C.); 2 Center for Translational Research on Autoimmune and Allergic Diseases-CAAD, Università del Piemonte Orientale, 28100 Novara, Italy

Osteopontin (OPN), a multifunctional protein, has emerged as a fascinating subject of study due to its diverse roles in various physiological and pathological processes. From bone remodeling to immune responses, OPN stands at the crossroads of multiple biological pathways, offering researchers a rich landscape for exploration.

This is mainly related to the fact that OPN is produced by a variety of cell types, serving as a molecular bridge connecting cells with their extracellular environment [1]. Moreover, it interacts with a spectrum of cell surface receptors, including integrins, the CD44 molecule [2], and the induced costimulatory molecule ligand (ICOSL) [3], underscoring its active involvement in fundamental cellular processes such as adhesion, migration, inflammation, and signaling pathways, among others. OPN exists in different isoforms and several post-translational modifications may change its properties. These include phosphorylation, glycosylation, and sulfation [4], as well as the proteolytic cleavage of full length OPN by enzymes like thrombin and matrix metalloproteinases, which produce OPN fragments, alter its biological roles, and demonstrate the gain of functions [5,6]. Lastly, two translational isoforms of OPN—secreted OPN (sOPN) and intracellular OPN (iOPN)—exist, each engaging in different functions due to their distinct cellular localizations. iOPN serves as an adaptor or scaffold protein in cellular signaling pathways and stabilizes intracellular proteins [7], while sOPN interacts with its various receptors and binding partners, resulting in cell- and context-dependent signaling [8].

In physiological contexts, OPN contributes significantly to tissue maintenance, wound healing, immune system regulation, and stress response mechanisms [9]. However, its involvement extends to the pathogenesis of several conditions, including atherosclerosis [10], cancer [11], autoimmune disorders [12], chronic inflammation [13], and sepsis [14]. OPN additionally functions as a diagnostic and prognostic biomarker for these disorders [15,16,17]. Lastly, OPN polymorphisms have been identified as significant factors linked to the susceptibility or progression of these diseases [18,19,20]. 

This Special Issue of *Biomedicines* compiled scientific evidence aimed at further unraveling the multifaceted roles of OPN in health and diseases, using a multidisciplinary approach, highlighting its significance and various functions in different biological contexts. The Special Issue encompasses eight papers. Three delve into the role of OPN as a biomarker for glomerulopathies and coronary artery disease, while two elucidate its involvement in melanoma metastasis and dental biofilm formation. Additionally, three reviews explore the multifaceted nature and versatility of OPN. 

The intricate interplay between OPN and microbial cells, especially bacteria, remains a largely unexplored realm. Kristensen et al. demonstrated the efficacy of bovine milk OPN in hindering the adhesion of three key bacteria responsible for dental biofilm formation, utilizing saliva-coated surfaces under shear-controlled flow conditions that mimicked the oral cavity. While this research provides valuable insights, further investigations are warranted to ascertain the broader applicability of these findings to other bacterial species commonly found in oral biofilms [21].

Broadening the horizon of OPN, Moszczuk et al. identified urine OPN as a potential biomarker in nephrology, specifically in glomerulopathies such as immunoglobulin A nephropathy (IgAN), membranous nephropathy (MN), and lupus nephritis (LN). Machine learning techniques were employed to correlate OPN levels with other clinical parameters and classify patients by glomerulopathy type. The resulting algorithm achieved 87% accuracy in differentiating IgAN from other glomerulopathies using urinary OPN levels. The effectiveness of OPN as a biomarker for discriminating MN and LN was less pronounced, possibly due to the smaller number of patients and the phenotypic heterogeneity of these conditions [22]. 

Larsson et al. evaluated both urinary and plasmatic levels of OPN in a cohort of healthy controls, examining their associations with various chemokines, cytokines, and growth factors. The results from the multivariate model indicated that plasmatic OPN levels were involved in a protein–protein interaction network linked to immune system regulation, apoptosis, and intracellular signal transduction. On the contrary, urinary OPN levels did not exhibit a similar involvement in these pathways [23]. 

By focusing on melanoma, Raineri et al. delved into the role of ICOSL in melanoma metastasis in the preclinical mouse model and its interaction with its ligands such as OPN and ICOS. The same group previously demonstrated, for the first time, that OPN is the novel receptor of ICOSL and neither OPN nor ICOS compete for the binding to ICOSL. Interestingly, OPN’s triggering of ICOSL reduced tumor growth while inducing angiogenesis and promoting tumor metastasis in the orthotopic 4T1 mammary carcinoma model [3]. The main findings of their latest work showed that the OPN produced by the tumor microenvironment (TME) interacts with stromal ICOSL, promoting melanoma metastasis; this interaction is inhibited by ICOS expressed on TME through the promotion of regulatory T cell expansion. The authors also demonstrated that the OPN-ICOSL interaction is increased in human melanoma metastasis compared to primary tumor [24].

Further enriching the multifaceted landscape of OPN genetics, Pérez-Hernández et al. evaluated the association of two OPN gene polymorphisms with premature coronary artery disease (pCAD), cardiovascular risk factors, and cardiometabolic parameters in Mexican individuals. While both polymorphisms exhibited similar allele and genotype frequencies in patients with pCAD and controls, patients showed an association with a high risk of hypoadiponectinemia, which is a significant predictor of endothelial dysfunction in both peripheral and coronary arteries. In the control group, there was an association with a high risk of elevated apolipoprotein B, which is related to dyslipidemia processes, and it is considered a significant predictor of cardiovascular diseases such as myocardial infarction [25].

Finally, this Special Issue included three reviews: the first examined the role of sOPN by different cell types in the central nervous system (CNS), comprising microglia, showing that it is upregulated in neurodegenerative and neuroinflammatory conditions. The understanding of the interplay between microglia and OPN is important for developing therapeutic interventions for CNS diseases [26]. The second underlined that OPN functions as a master regulator, orchestrating epithelial–mesenchymal plasticity in thyroid cancer through the modulation of the PI3K-AKT and MAPK signaling pathways where it induces the expression of transcription factors related to epithelial–mesenchymal transition [27]. The third review explored the significance of both full-length OPN and its protease-cleaved products across various infectious diseases, emphasizing the crucial role of OPN as a biomarker for infectious conditions. The inhibition of cleavage or the activities of these cleaved products may emerge as a potential strategy to enhance therapeutic outcomes for infectious diseases [5]. 

In conclusion, OPN remains a central figure in a spectrum of physiological and pathological processes. Through ongoing research endeavors, OPN has proven to be a key participant in a wide array of biological activities, encompassing vital roles in maintaining tissue integrity, regulating immune responses, and influencing complex processes in both health and disease. As investigations progress, OPN’s significance continues to unravel, shedding light on its involvement in diverse physiological functions and its impact on the progression of various pathological conditions. The expanding body of knowledge surrounding OPN also underscores its pivotal role in shaping our understanding of the intricate interplay within biological systems.
